# Supplementary material for: Increased levels of endogenous retroviruses trigger fibroinflammation and play a role in kidney disease development
Source: Nat Commun. 2023 Feb 2;14:559. doi: 10.1038/s41467-023-36212-w (PMC9895454; doi:10.1038/s41467-023-36212-w)
Supplement: Supplementary file 1 — Supplementary Information [file 41467_2023_36212_MOESM1_ESM.pdf]

Supplementary Figure 1

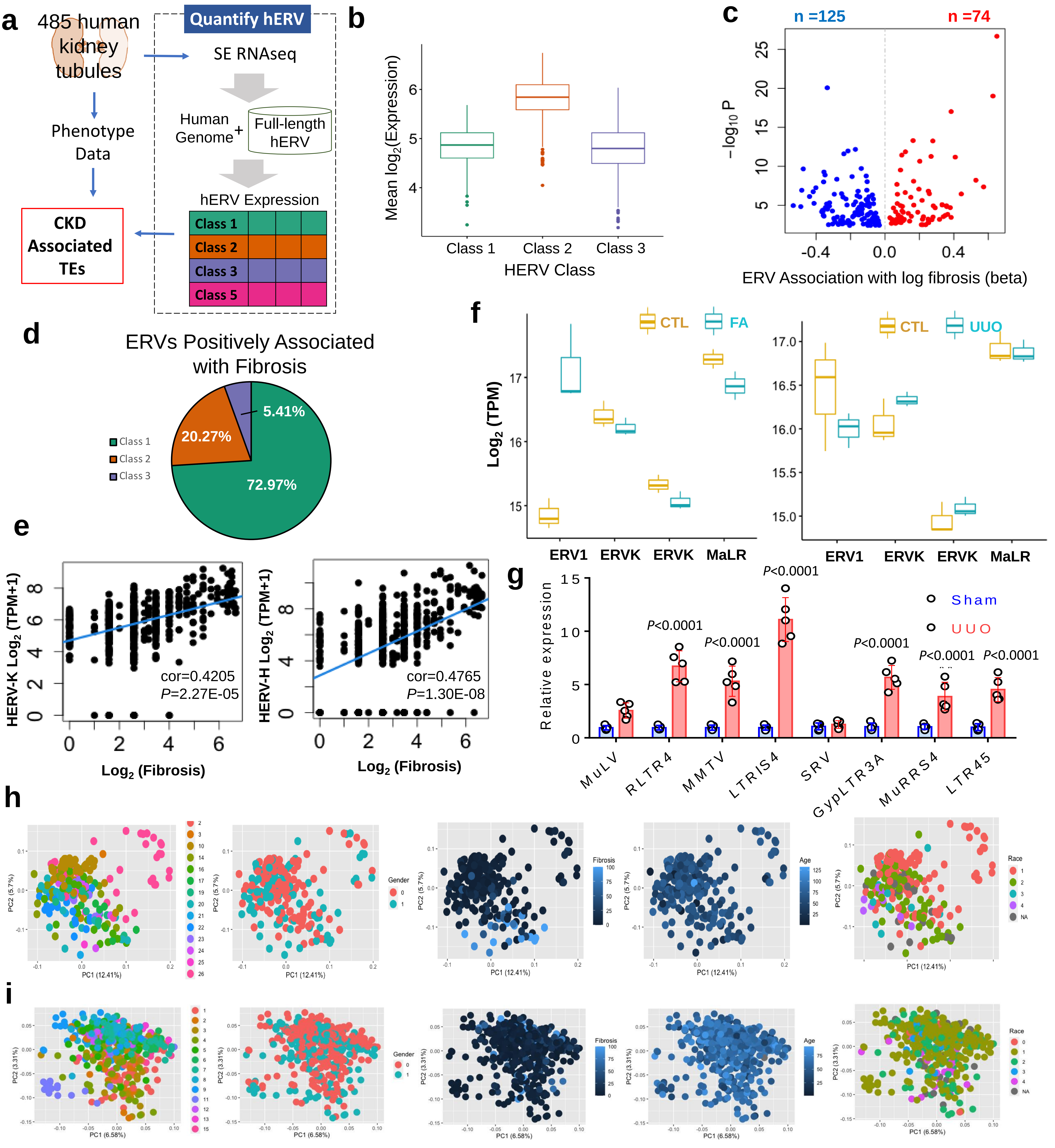

Supplementary Figure 1. Endogenous retroviral (ERV) expression and their association with kidney disease severity in patients and mice.

(a) Our quantification approach for full-length ERV expression in 485 human kidney tubule samples. (**b)** Boxplot representing the Mean Log_2 expression of ERV classes (*n* = 485 samples). Center lines show the medians; box limits indicate the 25th and 75th percentiles; bottom whiskers indicate 5th percentile and top whiskers indicate 95th percentile **(c)** Volcano plot of ERVs showing association with kidney fibrosis in human kidney samples. X-axis shows the beta values in the linear regression model (adjusted for age, sex, race, presence of hypertension, and diabetes). Y-axis shows the statistical significance (negative log p-value). Red dots show higher ERV and blue dots lower TEs (FDR < 0.05) in fibrosis. **(d)** Percentage of fibrosis-associated ERVs in each class. **(e)** The relationship between Endogenous Retrovirus K (ERVK), Endogenous Retrovirus 1 (ERVH), levels (log_2 TPM counts, x-axis), and the degree of kidney fibrosis (y-axis, percent fibrosis). Pearson correlation is shown. Student's t-test based on the Pearson correlation coefficient was used to calculate the statistical significance of the association. **(f)** Expression level of Endogenous retrovirus 1 (ERV1), Endogenous retrovirus K (ERVK), Endogenous retrovirus L (ERVL), and Mammalian apparent LTR Retrotransposons (MaLR), ERV classes in control (CTL, yellow) and folic acid (FA, blue) mice (left) (*n* = 6 in each). Expression level of ERV1, ERVK, ERVL, and MaLR ERV classes in Control (CTL, yellow) and Unilateral Ureteral Obstruction (UUO, blue) mice (right) (*n* = 6 in each). Center lines show the medians; box limits indicate the 25th and 75th percentiles; bottom whiskers indicate 5th percentile and top whiskers indicate 95th percentile **(g)** Relative levels of TE fragments (*MuLV, RLTR4, MMTV, LTRIS4, SRV, GypLTR3A, MURSS4.* and *LTR45*) in kidneys of UUO (red) mice compared to SHAM (blue) mice (*n* = 6 in each). Data were presented as the mean ± s.e.m and analyzed by two-tailed unpaired Student's t-test. Source data are provided as a Source Data file. **(h)** Principal component analysis of TEs in 240 human kidney samples batch, gender, fibrosis, age, and race (from left to right) highlighted. **(i)** Principal component analysis of ERVs in 485 human kidney samples batch, gender, fibrosis, age, and race (from left to right) highlighted.

Supplementary Figure 2

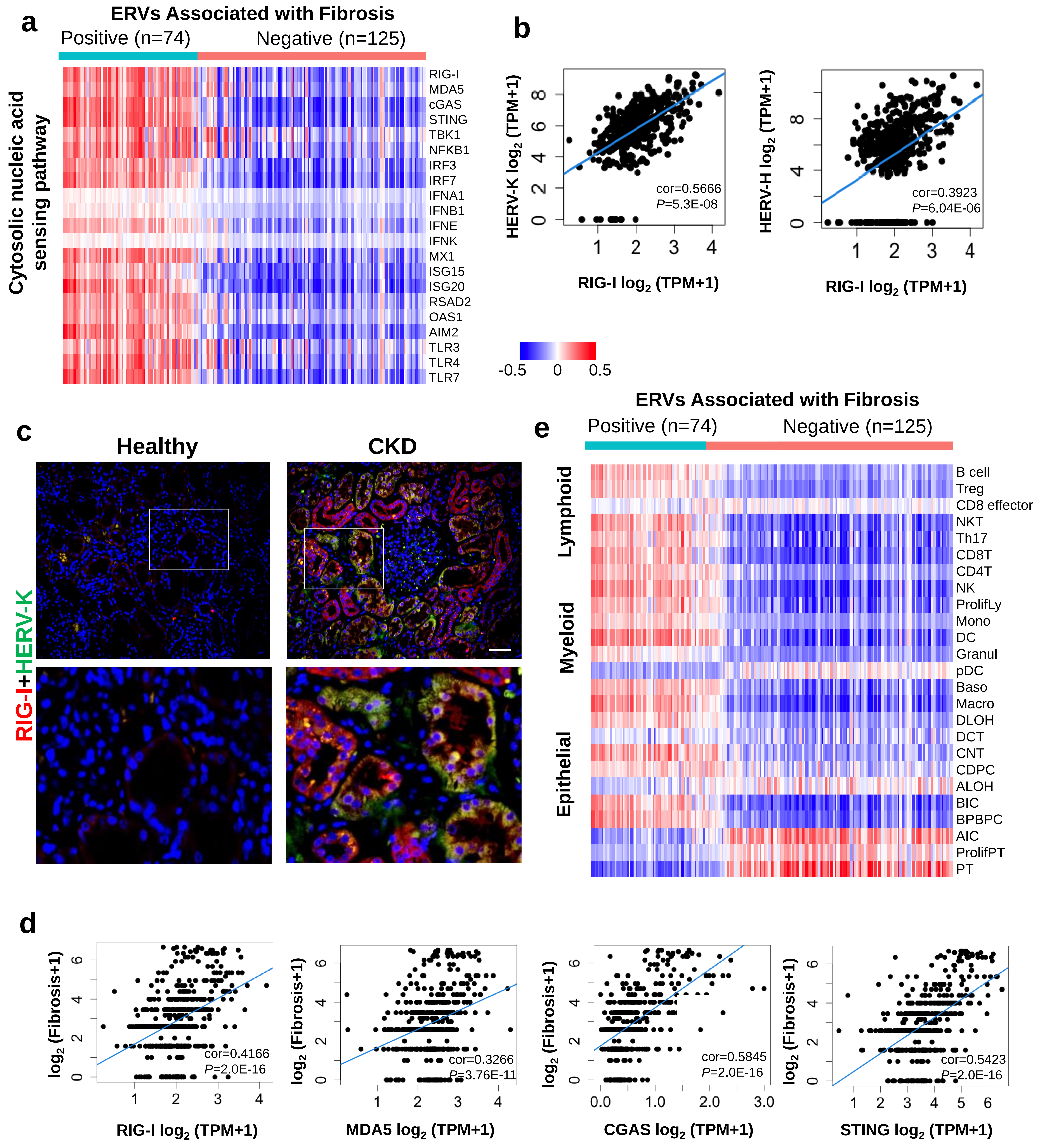

Supplementary Figure 2. Kidney HERV expressions correlate with nucleotide sensors and immune cell fractions.

(a) Heatmap of transposable elements (ERVs) (Pearson correlation coefficient) and cytosolic nucleic acid sensing pathway gene expression in human kidney samples. Each column is one ERV expression, and each row is one gene expression. Red indicates positive and blue indicates negative correlation coefficient. **(b)** Relationship between HERV-K and HERV-H with *RIG-I* $\log_2$ expression in human kidney samples. Pearson correlation is shown. Student's *t*-test based on the Pearson correlation coefficient was used to calculate the statistical significance of the association. **(c)** Representative double immunofluorescence images of RIG-I (Red) with HERV-K (green) in healthy and CKD human kidneys. Scale bar, 10 μm. Data are representative of two independent experiments. **(d)** Correlation between *RIG-I, MDA5, CGAS,* and *STING* $\log_2$ transcript levels and $\log_2$ fibrosis score in 485 human kidney tissue samples. Pearson correlation is shown. Student's t-test based on the Pearson correlation coefficient was used to calculate the statistical significance of the association **(e)** Heatmap of ERV (Pearson correlation coefficient) and kidney cell fractions in human kidney samples. Each column is one TE expression, and each row is one gene expression. Red indicates positive and blue indicates negative correlation. CD8T, CD8 T cells; CD4T, CD4 T cells; NK, natural killer cells; ProlifLy, proliferating lymphocytes; Mono, monocytes; DC, dendritic cells; Granul, granulocytes; pDC, plasmacytoid dendritic cells; Baso, basophils; Macro, macrophages; DLOH, descending loop of Henle; DCT, distal convoluted tubule; CNT, connecting tubule cells; CDPC, ALOH, loop of Henle; BIC, type-B intercalated cells; BPBPC, peripheral blood progenitor cells; AIC, type-A intercalated cells; ProlifPT, proliferating proximal tubules; PT, proximal tubules.

Supplementary Figure 3

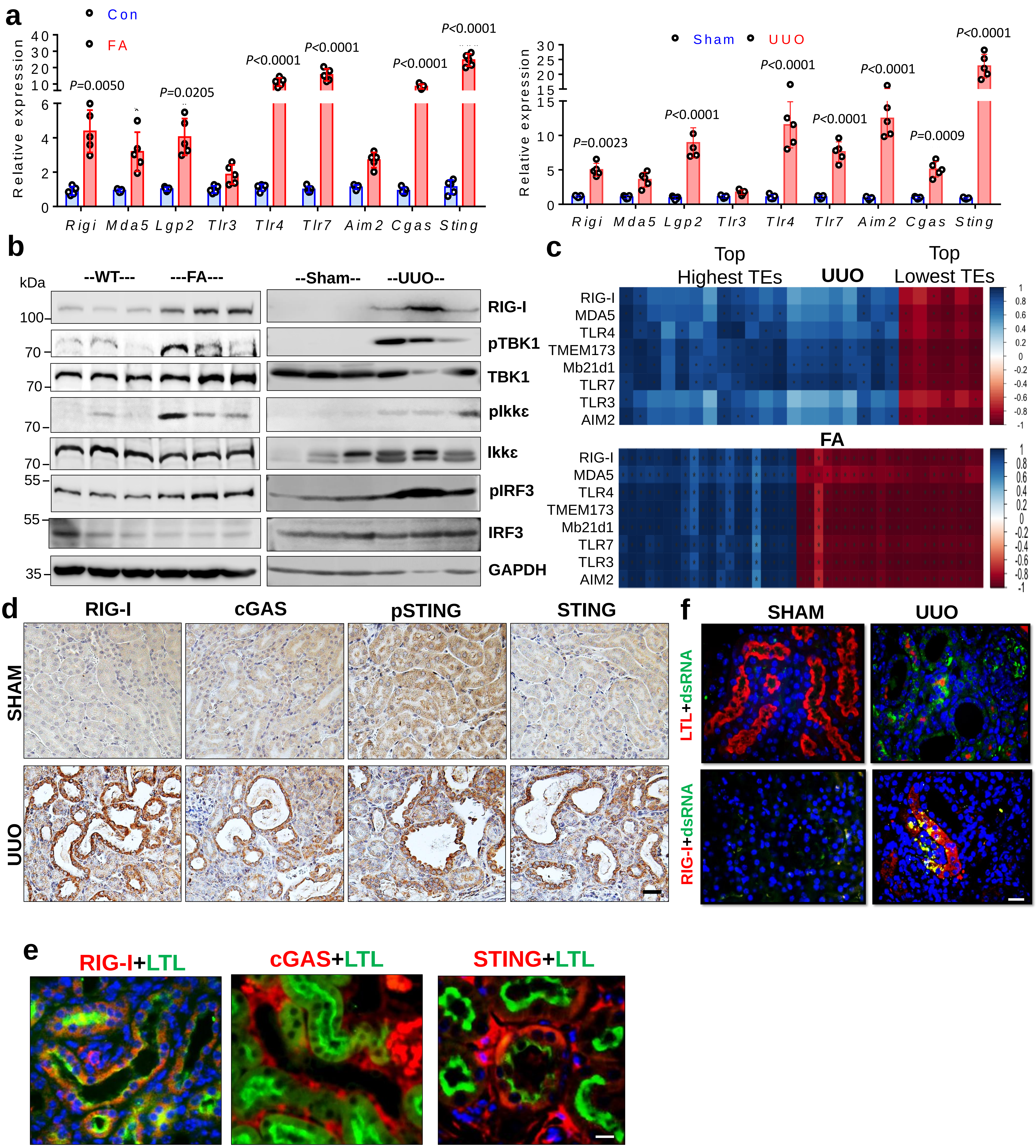

Supplementary Figure 3. Increased nucleotide sensors level strongly correlate with ERVs expression in mice kidneys. **(a)** Relative mRNA levels of cytoplasmic nucleic acid sensors (*Rigi, Mda5, Lgp2, Tlr3, Tlr4, Tlr7, Aim2, Cgas,* and *Sting*) in FA-induced (red) and UUO mice kidney fibrosis model (red) as compared to control (black) (n=5 in each). Data were presented as the mean ± s.e.m and analyzed by two-tailed unpaired Student's t-test. **(b)** Western blot showing the representative images of RIG-I, pTBK1, TBK1, pIKKe, Ikke, pIRF3, and IRF3 protein levels in FA-induced (left) and UUO (right) mice kidney fibrosis model. GAPDH was used as a loading control. **(c)** Heatmap of TE Pearson correlation coefficient in mouse kidney samples. Each column is one kidney sample, and each row is one TE expression. Red indicates higher and blue indicates lower Pearson correlation coefficient. **(d)** Representative images of IHC of RIG-I, cGAS, pSTING, and STING protein expression in kidneys of SHAM and UUO mice. Scale bar, 10 μm. Data are representative of two independent experiments. **(e)** Representative double immunofluorescence images of RIG-I, cGAS, and STING (red) with PT marker: LTL (green) in kidney sections of UUO mice. Scale bar, 10 μm. *Levels of RIG-I, cGAS, and STING were too low to detect by IF in the control kidney section. **(f)** Representative double immunofluorescence images of dsRNA (green) with PT marker: LTL (red) (upper panel) and co-staining of RIG-I (red) with dsRNA (green) (lower panel) in kidney sections of SHAM and UUO mice. Scale bar, 5 μm. Data are representative of two independent experiments (**d-f**). Source data are provided as a Source Data file.

Supplementary Figure 4

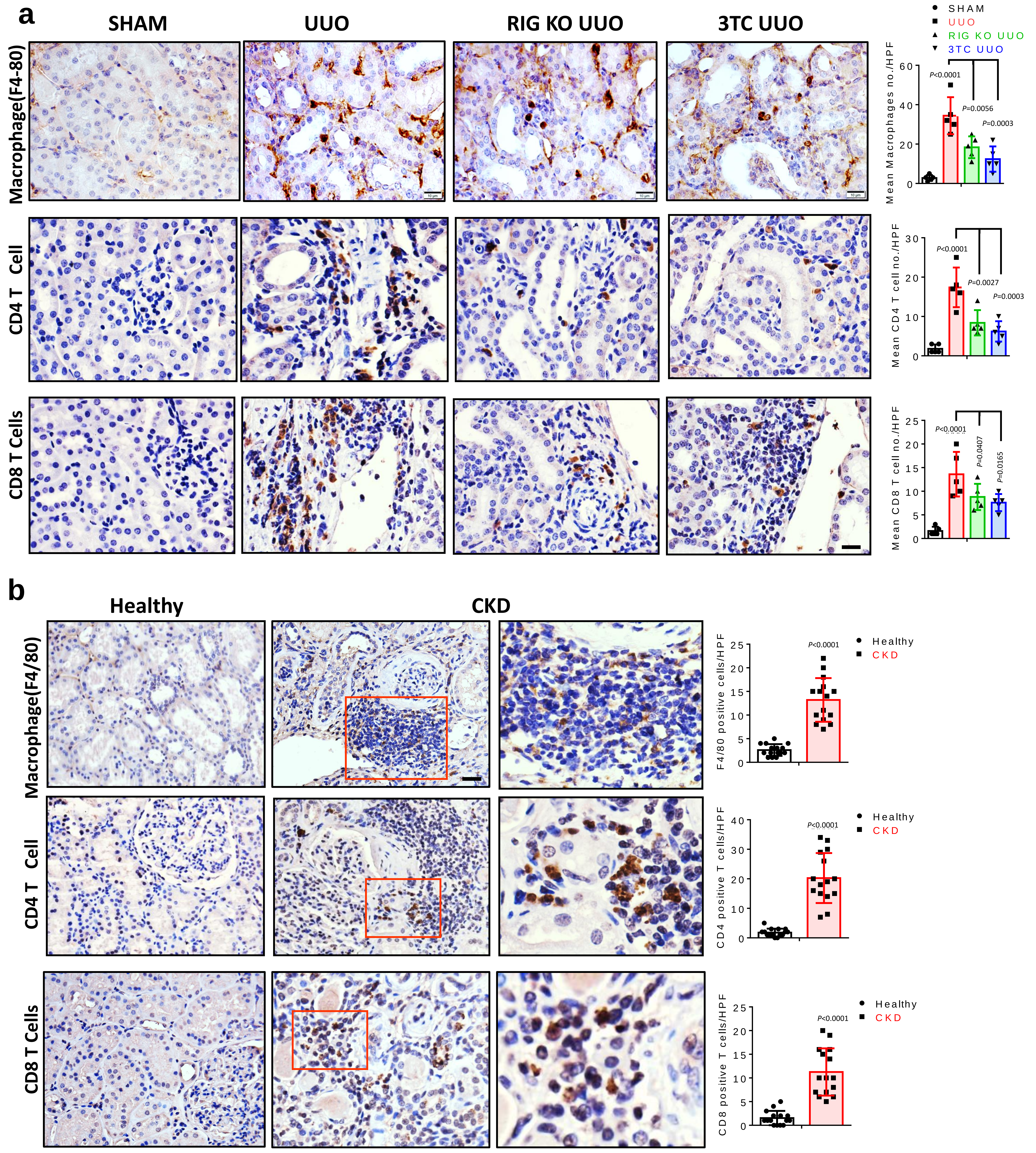

Supplementary Figure 4: Immune cells in mouse and human kidney disease.

(a) Representative images of IHC of F4-80 (macrophage marker), CD4 T cell, and CD8 T cell staining and respective quantification in kidneys of SHAM (black), UUO (red), RIG KO UUO (green), and 3TC UUO (blue) mice. Scale bar, 10 μm. Average no. of F4-80/CD4/CD8 cells per mouse were plotted as mean F4-80/CD4/CD8 cells/(high power field) HPF ± s.e.m. (*n* = 5 in each) and analyzed using a one-way ANOVA followed by Tukey post hoc test for multigroup comparison. **(b)** Representative images of IHC of F4-80, CD4 T cell, and CD8 T cell staining and respective quantification in healthy (black) and CKD (red) human kidneys. Scale bar, 10 μm. Average no. of F4-80/CD4/CD8 cells per 15 fields were quantified and plotted as mean ± s.e.m. (*n* = 3 in each) and were analyzed using a two-tailed Student's t-test. Source data are provided as a Source Data file.

Supplementary Figure 5

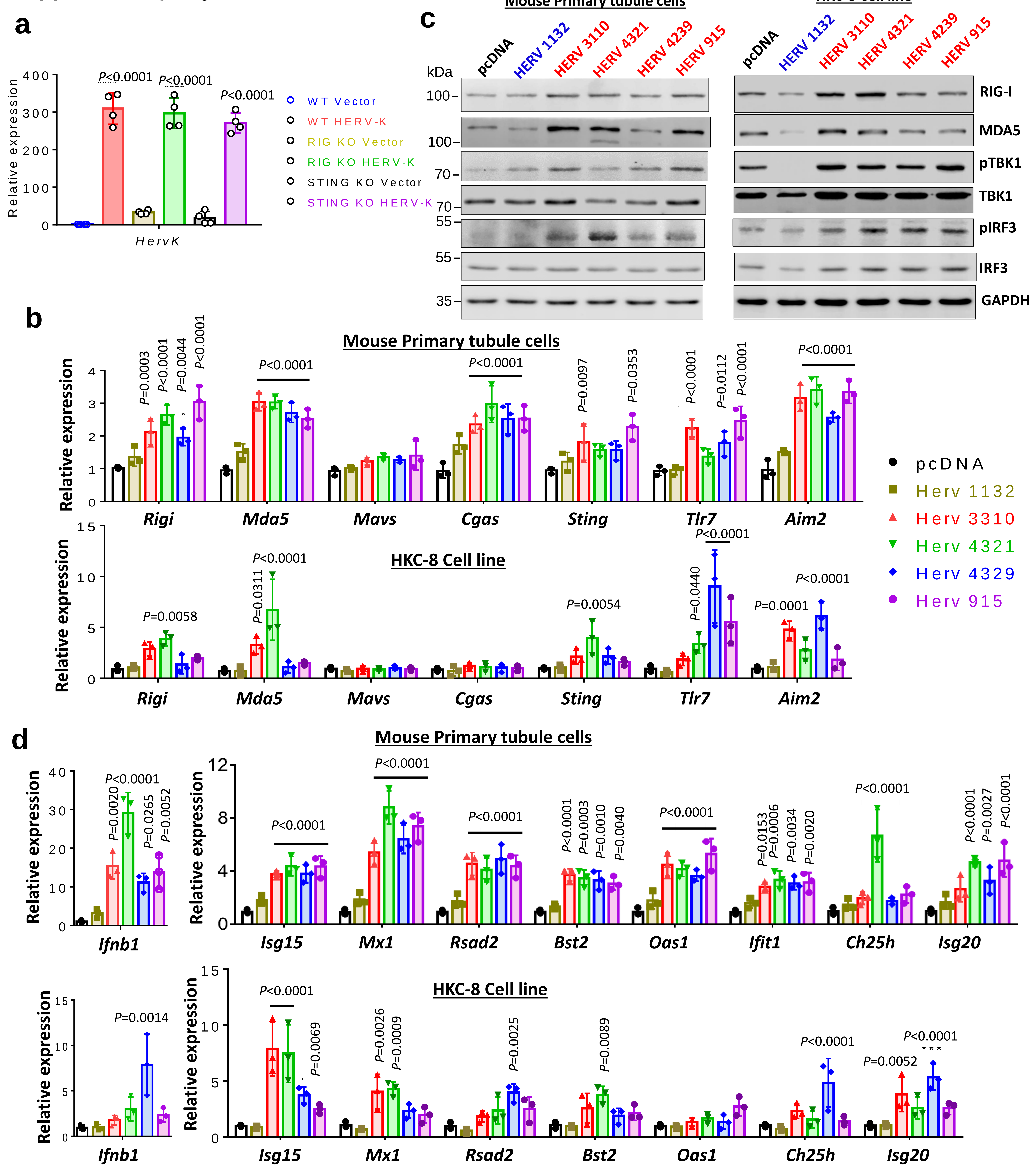

Supplementary Figure 5. HERV RNA activates RNA sensing pathways in PT cells.

(a) The relative RNA level of *HERV-K* in WT (Vector: blue, HERV-K: red), RIG KO (Vector: yellow, HERV-K: green), and STING KO (Vector: black, HERV-K: magenta) PTECs transfected with HERV-K/Vector (*n* = 4 in each). **(b)** RNA levels of cytosolic nucleotide sensors (*Rigi, Mda5, Mavs, cGas, Sting, Tlr7*, and *Aim2*) in PT cells and HKC-8 cells transfected with HERV/pcDNA in vitro transcribed RNA for 12 hours (*n* = 3 in each). **(c)** Representative images of protein levels of RIG-I, MDA5, pTBK1, TBK1, pIRF3, and IRF3 in PT cells and HKC-8 cells transfected with HERV RNA/pcDNA in vitro transcribed RNA for 12 hours. GAPDH was used as a loading control. Data are representative of two independent experiments. **(d)** RNA levels of *Ifnb* and ISGs (*Isg15, Mx1, Rsad2, Bst2, Oas1, Ifit1, Ch25h,* and *Isg20*) in PT cells and HKC-8 cells transfected with HERV/pcDNA in vitro transcribed RNA for 12 hours (*n* = 3 in each). Data are represented as mean ± s.e.m. and analyzed using a one-way ANOVA followed by Tukey post hoc test for multigroup comparison (**a-b**, **d**). Source data are provided as a Source Data file.

Supplementary Figure 6

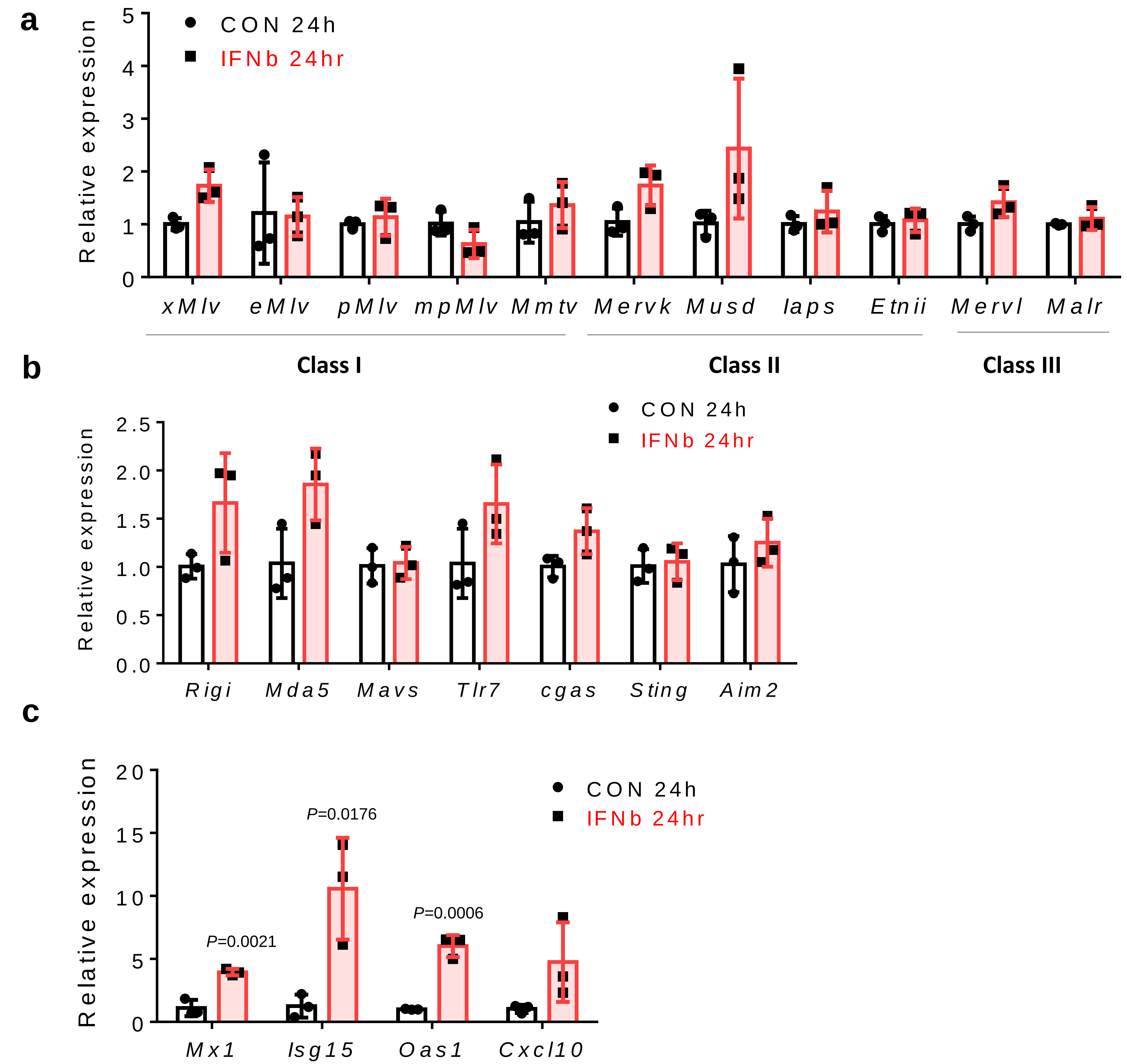

Supplementary Figure 6. IFN is not the main regulator of ERVs expression in PT cells.

(a) Relative expression levels of Class I ERVs (*xMlv, eMlv, pMlv, mpMlv,* and *Mmtv*), Class II (*MervK, Musd, Iaps,* and *Etnii*), and Class III (*Mervl* and *Malr*) in PTECs post 24 hours treatment with mIFNβ (red) or PBS (black) (*n* = 3 in each). **(b)** Relative mRNA levels of cytoplasmic nucleic acid sensors (*Rigi, Mda5, Mavs, Tlr7, Cgas, Sting,* and *Aim2)* in PTECs following treated with mIFNβ for 24 hours ((*n* = 3 in each). **(c)** RNA levels of IFN-stimulated genes (*Mx1, Isg15, Oas1,* and *Cxcl10*) in PTECs treated following mIFNβ treatment for 24 hours ((*n* = 3 in each). Data were presented as the mean ± s.e.m and analyzed by two-tailed unpaired Student's t-test. Source data are provided as a Source Data file.

Supplementary Figure 7

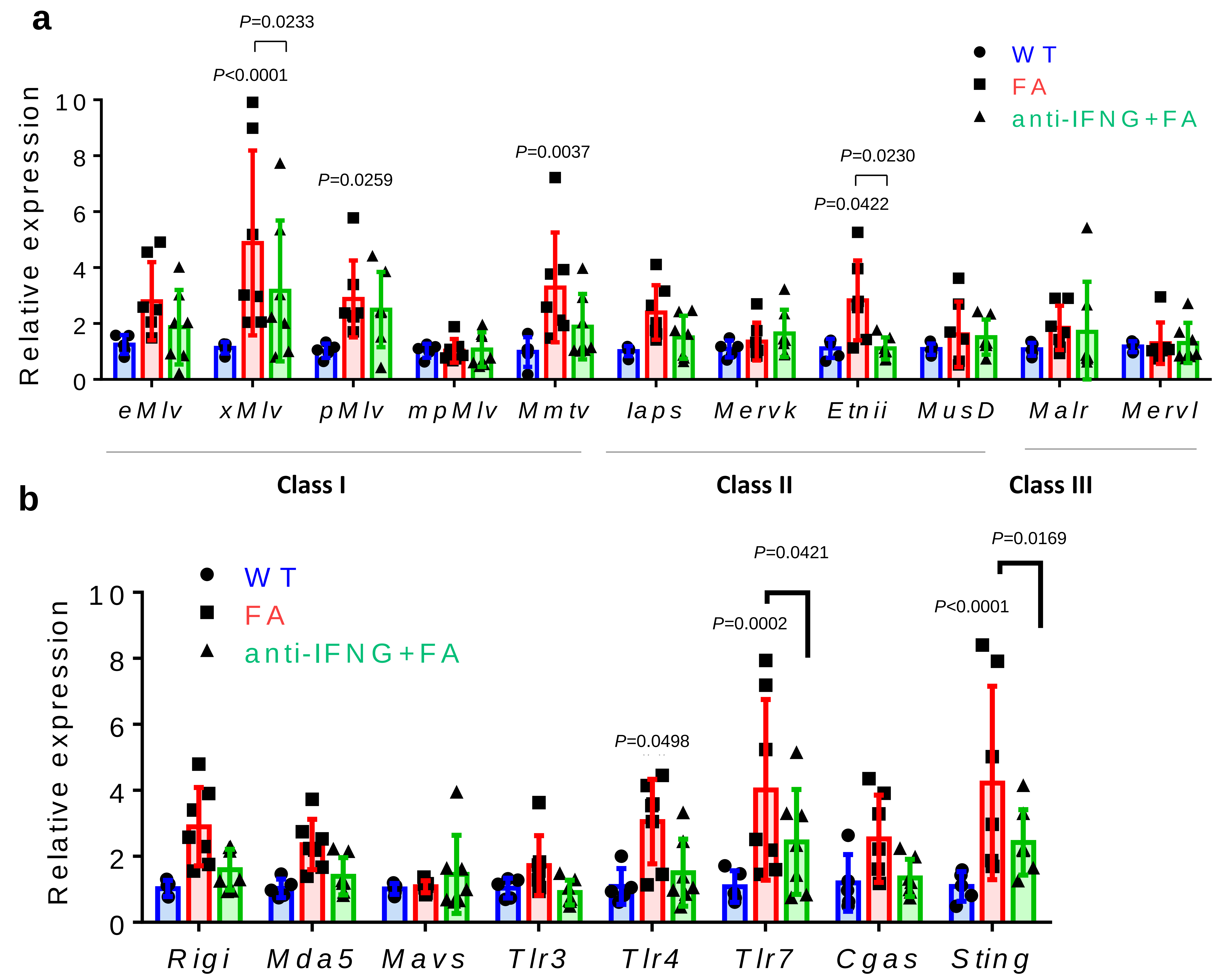

Supplementary Figure 7. ERVs levels in mice treated with IFNG neutralizing antibody.

(a) Relative expression levels of Class I ERVs (*eMlv, xMlv, pMlv, mpMlv,* and *Mmtv*), Class II (*Iaps*, *Mervk, Etnii,* and *MusD*), and Class III (*Malr* and *Mervl*) in WT (blue), FA (red), and anti-IFNG+FA (green) mice kidneys. **(b)** Relative mRNA levels of cytoplasmic nucleic acid sensors (*Rigi, Mda5, Mavs, Tlr3, Tlr4, Tlr7, Cgas,* and *Sting)* in WT, FA, and anti-IFNG+FA mice kidneys. {WT, n = 5; FA and anti-IFNG+FA, n = 7 in each (**a-b**)}. Data are represented as mean ± s.e.m. and analyzed using a two-way ANOVA followed by Tukey post hoc test for multigroup (**a-b**). Source data are provided as a Source Data file.

Supplementary Figure 8

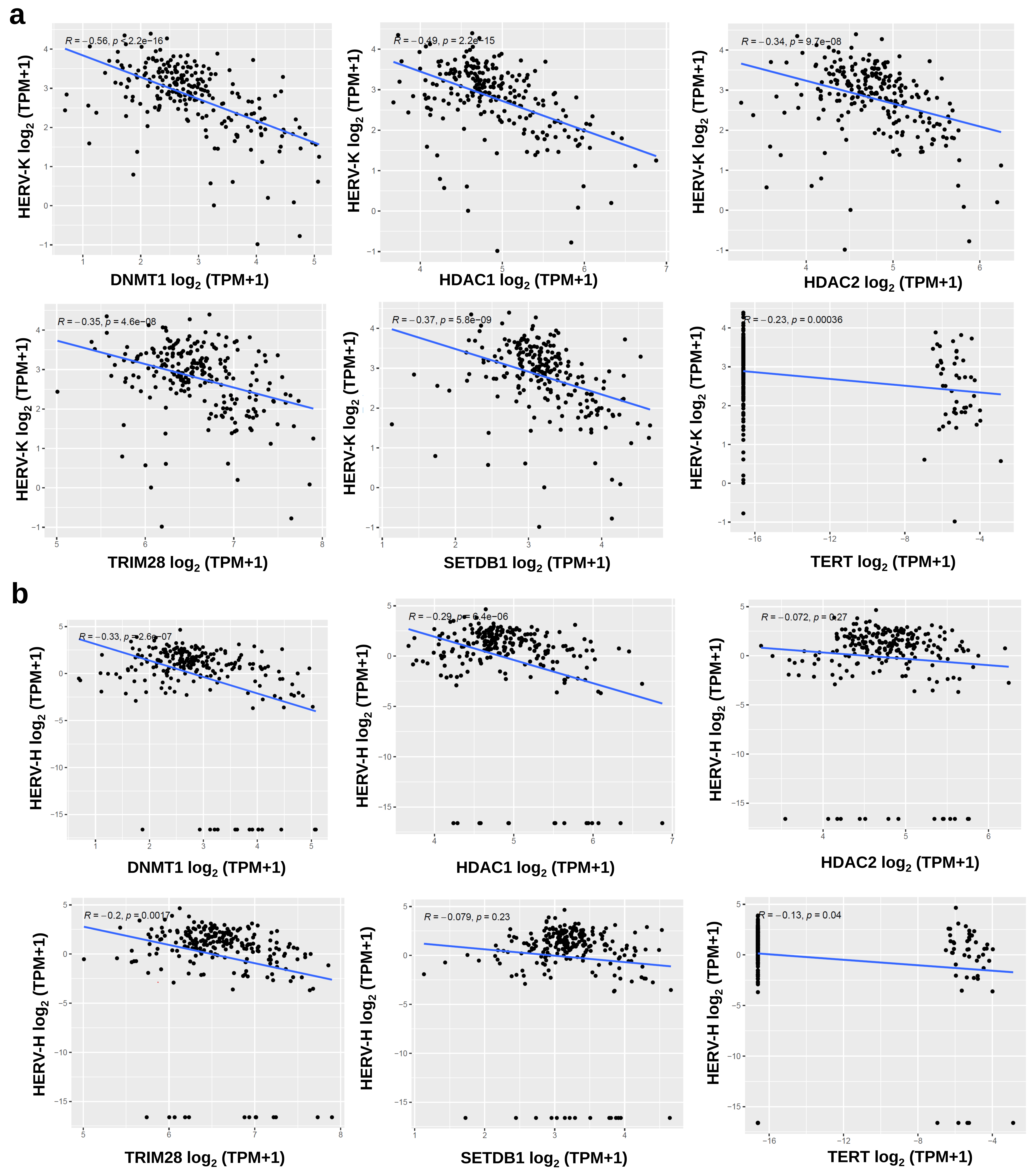

Supplementary Figure 8: Correlation of HERV-K and HERV-H expression with epigenetic regulators in human kidneys.

(a) The relationship between levels of Human Endogenous Retrovirus K (HERV-K) (log_2 TPM counts) and epigenetic regulators (*DNMT1, HDAC1, HDAC2, TRIM28,* and *SETDB1)* and *TERT* log_2 expression in human kidney samples. **(b)** The relationship between levels of Human Endogenous Retrovirus H (HERV-H) (log_2 TPM counts) and epigenetic regulators (*DNMT1, HDAC1, HDAC2, TRIM28,* and *SETDB1)* and *TERT* log_2 expression in human kidney samples. Pearson correlation is shown. Student's *t*-test based on the Pearson correlation coefficient was used to calculate the statistical significance of the association (**a-b**).

Supplementary Figure 9

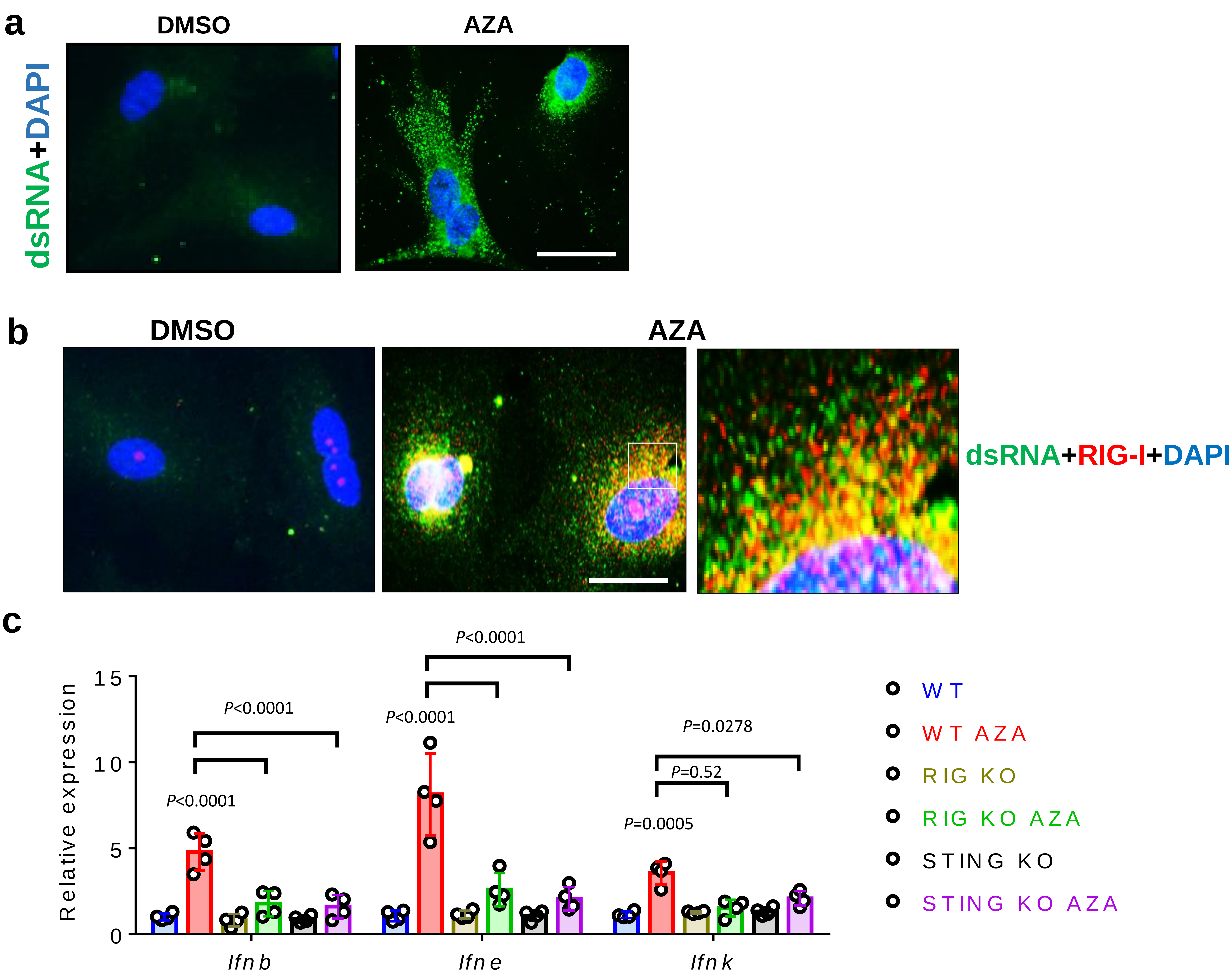

Supplementary Figure 9. Increased ERVs expression triggers IFN response in PTECs following AZA (DNMTi) treatment. **(a)** Representative immunofluorescence images of dsRNA staining (green) and DAPI (blue) in WT PTECs cells treated with AZA or DMSO. Scale bar, 10μM. Data are representative of three independent experiments **(b)** Representative immunofluorescence images of dsRNA(green) and RIG-I (red) in WT PTECs cells treated with AZA or DMSO. Scale bar, 10μM. Data are representative of three independent experiments. **(c)** *Ifnb, Ifne,* and *Ifnk* RNA expression in WT (DMSO: blue, AZA: red), RIG KO (DMSO: yellow, AZA: green), and STING KO (DMSO: black, AZA: magenta) PTECs treated with AZA/DMSO (n = 4 in each). Data are represented as mean ± s.e.m. and analyzed using a one-way ANOVA followed by Tukey post hoc test for multigroup. Source data are provided as a Source Data file.

Supplementary Figure 10

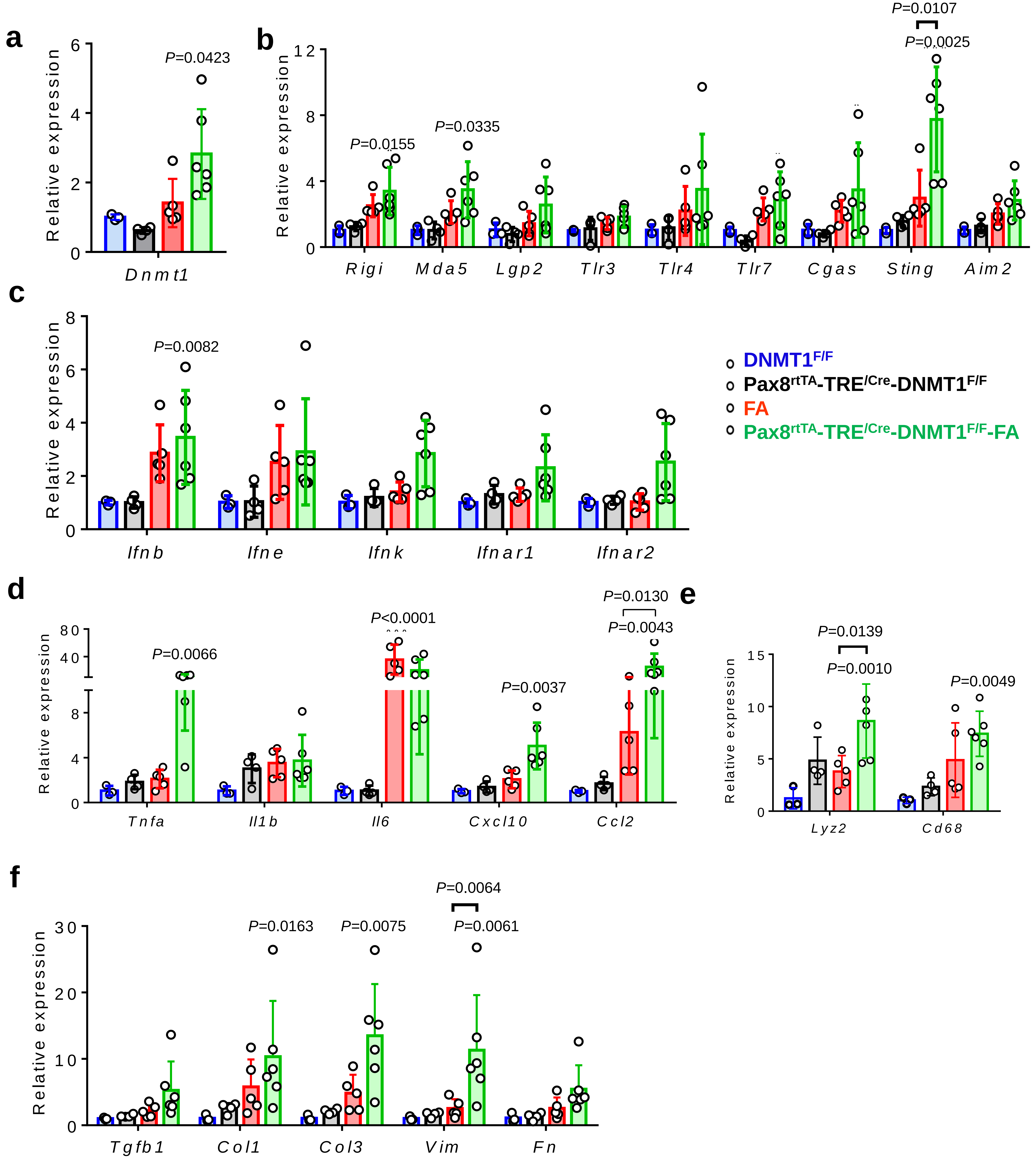

Supplementary Figure 10. Conditional inducible genetic deletion of DNMT1 is associated with increased TE and ERV expression and more severe injury.

(a) Relative mRNA levels of *Dnmt1* in kidneys of DNMT1$^{F/F}$ and Pax8^{rtTA}-TRE/^{Cre}-DNMT1$^{F/F}$ mice injected with FA. (DNMT1$^{F/F}$, blue; Pax8^{rtTA}-TRE/^{Cre}-DNMT1$^{F/F}$, black, FA, red; Pax8^{rtTA}-TRE/^{Cre}-DNMT1$^{F/F}$ FA, green). **(b)** Relative RNA levels of cytosolic RNA sensors (*Rigi, Mda5, Mavs, Tlr3, Tlr4,* and *Tlr7*) and DNA sensors *Cgas, Sting,* and *Aim2* in kidneys of DNMT1$^{F/F}$ and Pax8^{rtTA}-TRE/^{Cre}-DNMT1$^{F/F}$ mice injected with FA. **(c)** Relative mRNA levels of Type I IFN (*Ifnb, Ifne,* and *Ifnk*) and its receptors *Ifnar1/2* in kidneys of DNMT1$^{F/F}$ and Pax8^{rtTA}-TRE/^{Cre}-DNMT1$^{F/F}$ mice injected with FA. **(d)** Relative mRNA levels of proinflammatory cytokines (*Tnfa, Il1b, Il6, Cxcl10,* and *Ccl2*) in kidneys of DNMT1$^{F/F}$ and Pax8^{rtTA}-TRE/^{Cre}-DNMT1$^{F/F}$ mice injected with FA. **(e)** Expression of macrophage markers (*lyz2* and *Cd68*) in kidneys of DNMT1$^{F/F}$ and Pax8^{rtTA}-TRE/^{Cre}-DNMT1$^{F/F}$ mice injected with FA. **(f)** Expression of profibrotic markers (*Tgfb1, Col1, Col3, Vim,* and *Fn*) in kidneys of DNMT1$^{F/F}$ and Pax8^{rtTA}-TRE/^{Cre}-DNMT1$^{F/F}$ mice injected with FA. {(DNMT1$^{F/F}$, *n* = 3; Pax8^{rtTA}/TRE^{Cre}/DNMT1$^{F/F}$, *n* = 4; FA, *n* = 5; Pax8^{rtTA}/TRE^{Cre}/DNMT1$^{F/F}$ FA, *n* = 6 (**a-f**)}. Data are represented as mean ± s.e.m. and analyzed using a one-way ANOVA followed by Tukey post hoc test for multigroup (**a-f**). Source data are provided as a Source Data file.

Supplementary Figure 11

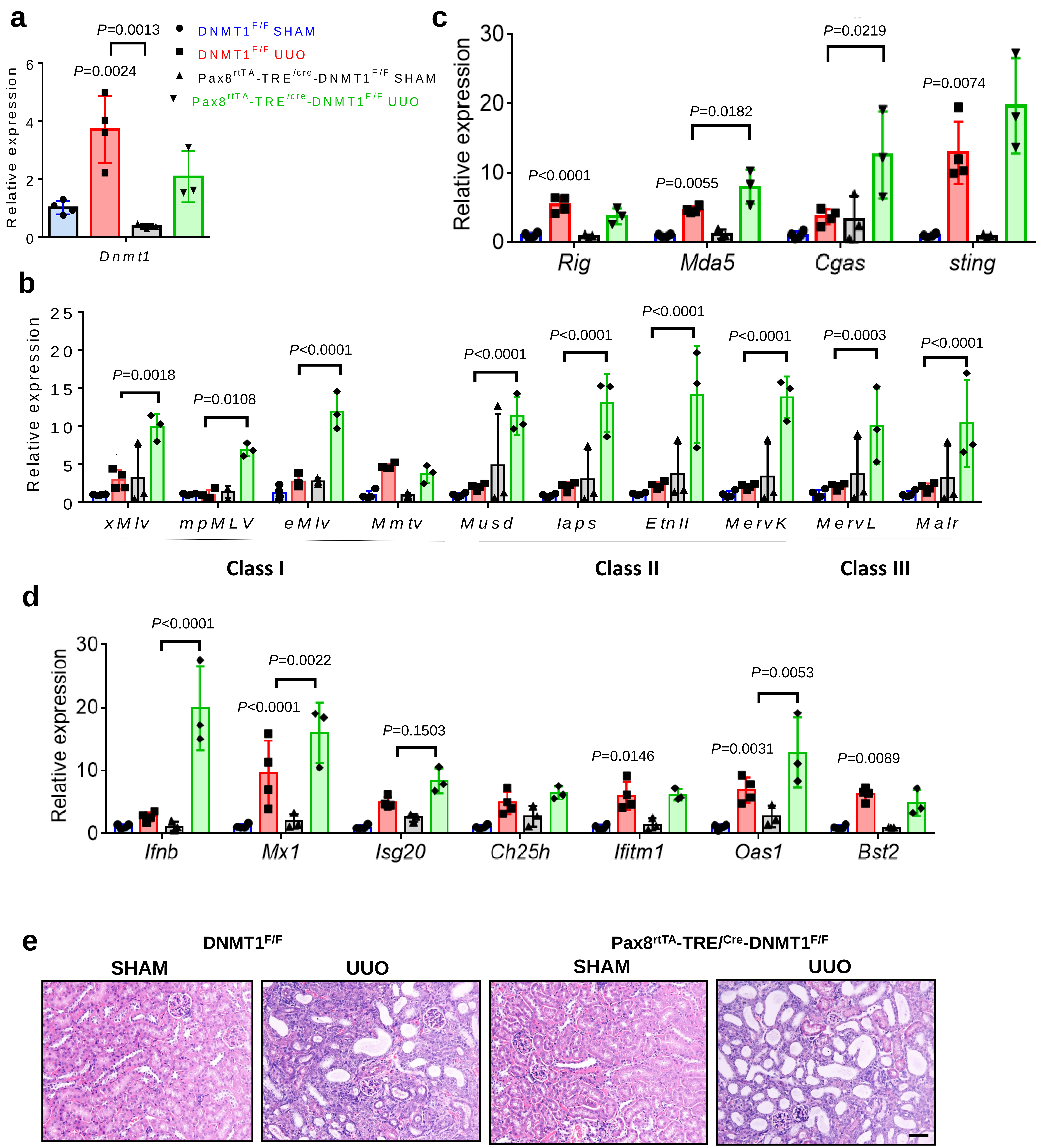

Supplementary Figure 11. Conditional inducible genetic deletion of DNMT1 in proximal tubule cells aggravates UUO-induced renal injury.

(a) Relative mRNA levels of *Dnmt1* in SHAM and UUO kidneys of $DNMT1^{F/F}$ and $Pax8^{rtTA}$-TRE/^{Cre}-$DNMT1^{F/F}$ mice. ($DNMT1^{F/F}$ SHAM, blue; $Pax8^{rtTA}$-TRE/^{Cre}-$DNMT1^{F/F}$, black; $DNMT1^{F/F}$ UUO, red; $Pax8^{rtTA}$-TRE/^{Cre}-$DNMT1^{F/F}$ UUO, green). **(b)** Relative mRNA levels of Class I ERVs (*xMlv, mpMlv, eMlv,* and *Mmtv*), Class II (*Musd, Iaps, EtnII* and *MervK*), and Class III (*Mervl* and *Malr*) in SHAM and UUO kidneys of $DNMT1^{F/F}$ and $Pax8^{rtTA}$-TRE/^{Cre}-$DNMT1^{F/F}$ mice. **(c)** Relative RNA levels of cytosolic RNA sensors (*Rigi, Mda5, Cgas,* and *Sting)* in SHAM and UUO kidneys of $DNMT1^{F/F}$ and $Pax8^{rtTA}$-TRE/^{Cre}-$DNMT1^{F/F}$ mice. **(d)** Relative mRNA level of *Ifnb* and ISGs (*Mx1, Isg20, Ch25h, Ifitm1, Oas1,* and *Bst2*) in SHAM and UUO kidneys of $DNMT1^{F/F}$ and $Pax8^{rtTA}$-TRE/^{Cre}-$DNMT1^{F/F}$ mice. {($DNMT1^{F/F}$ SHAM, $n = 4$; $DNMT1^{F/F}$ UUO, $n = 4$ $Pax8^{rtTA}$/TRE^{Cre}/$DNMT1^{F/F}$ SHAM, $n = 3$; $Pax8^{rtTA}$/TRE^{Cre}/$DNMT1^{F/F}$ UUO, $n = 3$ (**a-d**)}. Data are represented as mean ± s.e.m. and analyzed using a one-way ANOVA followed by Tukey post hoc test for multigroup (**a-d**). **(e)** Representative images of H and E staining of kidney sections from SHAM and UUO kidneys of $DNMT1^{F/F}$ and $Pax8^{rtTA}$-TRE/^{Cre}-$DNMT1^{F/F}$ mice. Scale bar: 10µM. Data are representative of two independent experiments. Source data are provided as a Source Data file.

Supplementary Figure 12

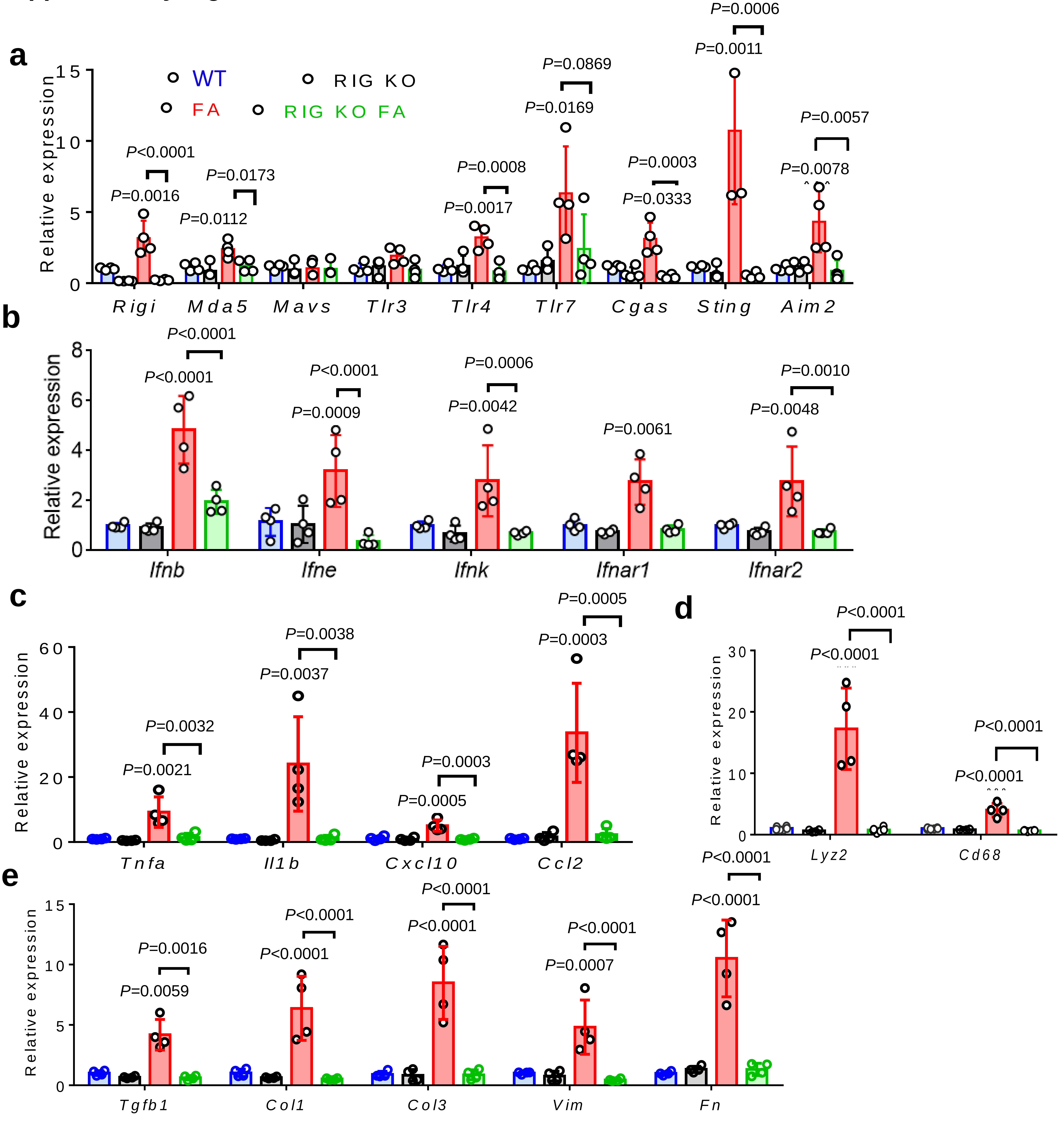

Supplementary Figure 12. RIG-I deficiency ameliorates FA-induced kidney fibrosis.

(a) Relative mRNA levels of cytosolic RNA sensors (*Rigi, Mda5, Mavs, Tlr3, Tlr4,* and *Tlr7*) and DNA sensors: *Cgas, Sting,* and *Aim2* in kidneys of WT and RIG KO mice injected with FA. (WT, blue; FA, red; RIG KO, black; RIG KO FA, green). **(b)** Relative mRNA levels of Type I IFN (*Ifnb, Ifne,* and *Ifnk*) and its receptors *Ifnar1/2* in kidneys of WT and RIG KO mice injected with FA. **(c)** Relative mRNA levels of proinflammatory cytokines (*Tnfa, Il1b, Cxcl10,* and *Ccl2*) in kidneys of WT and RIG KO mice injected with FA. **(d)** Relative mRNA levels of macrophage markers (*lyz2* and *Cd68*) in kidneys of WT and RIG KO mice injected with FA. **(e)** Profibrotic markers RNA levels (*Tgfb1, Col1, Col3, Vim,* and *Fn*) in kidneys of WT and RIG KO mice injected with FA. {WT and RIG KO, *n* =5 in each; FA and RIG KO FA, *n* =4 in each (**a-e**)}. Data are represented as mean ± s.e.m. and analyzed using a one-way ANOVA followed by Tukey post hoc test for multigroup (**a-e**). Source data are provided as a Source Data file.

Supplementary Figure 13

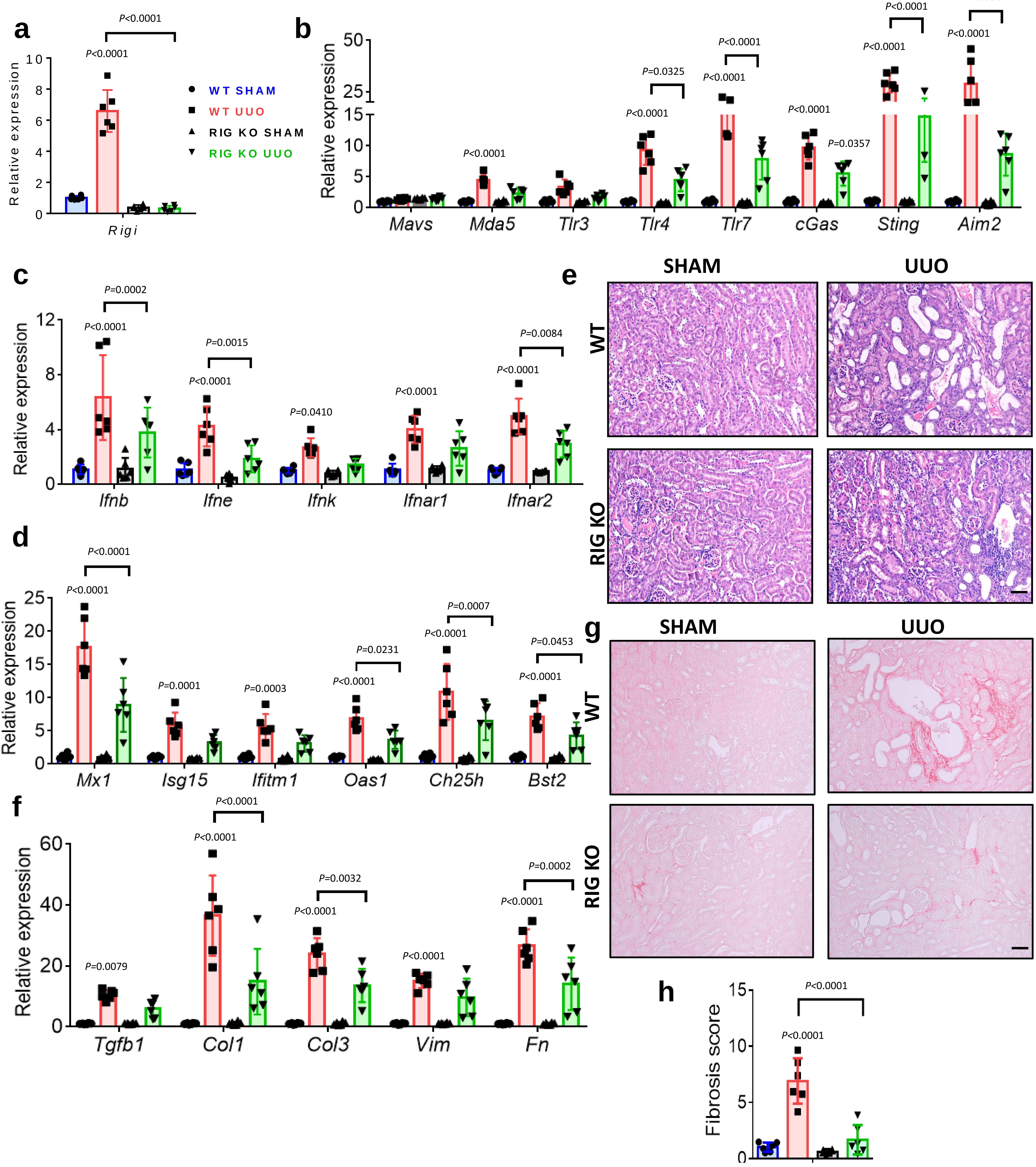

Supplementary Figure 13. Lower inflammation and kidney fibrosis in RIG KO mice post UUO injury.

(a) Relative mRNA levels of *Rigi* in SHAM and UUO kidneys of WT and RIG KO mice (WT SHAM, blue; WT UUO, red; RIG KO SHAM, black; RIG KO UUO, green) (*n* = 6 in each). **(b)** Relative RNA levels of cytosolic RNA sensors (*Mavs, Mda5, Tlr3, Tlr4, Tlr7, Cgas, Sting,* and *Aim2)* in SHAM and UUO kidneys of WT and RIG KO mice (*n* = 6 in each). **(c)** Relative mRNA level of ISGs (*Mx1, Isg15, Ifitm1, Oas1, Ch25h,* and *Bst2*) in SHAM and UUO kidneys of WT and RIG KO mice (*n* = 6 in each). **(d)** Relative mRNA level of IFNs (*Ifnb, Ifne,* and *Ifnk*) and its receptors (*Ifnar1* and *Ifnar2*) in SHAM and UUO kidneys of WT and RIG KO mice. **(e)** Representative images of H&E staining of kidney sections from SHAM and UUO kidneys of WT and RIG KO mice. Scale bar: 10μM. Data are representative of two independent experiments. **(f)** Relative mRNA level of fibrosis markers (*Tgfb1, Col1, Col3, Vim,* and *Fn*) in SHAM and UUO kidneys of WT and RIG KO mice (*n* = 6 in each). **(g)** Representative images of PS staining of kidney sections from SHAM and UUO kidneys of WT and RIG KO mice. Scale bar: 10μM. Data are representative of two independent experiments. **(h)** Quantification of tubulointerstitial fibrosis by Sirius red staining in SHAM and UUO kidney from WT and RIG KO mice (*n* = 6 in each). Data are represented as mean ± s.e.m. and analyzed using a one-way ANOVA followed by Tukey post hoc test for multigroup (**a-d, f, h**). Source data are provided as a Source Data file.

Supplementary Figure 14

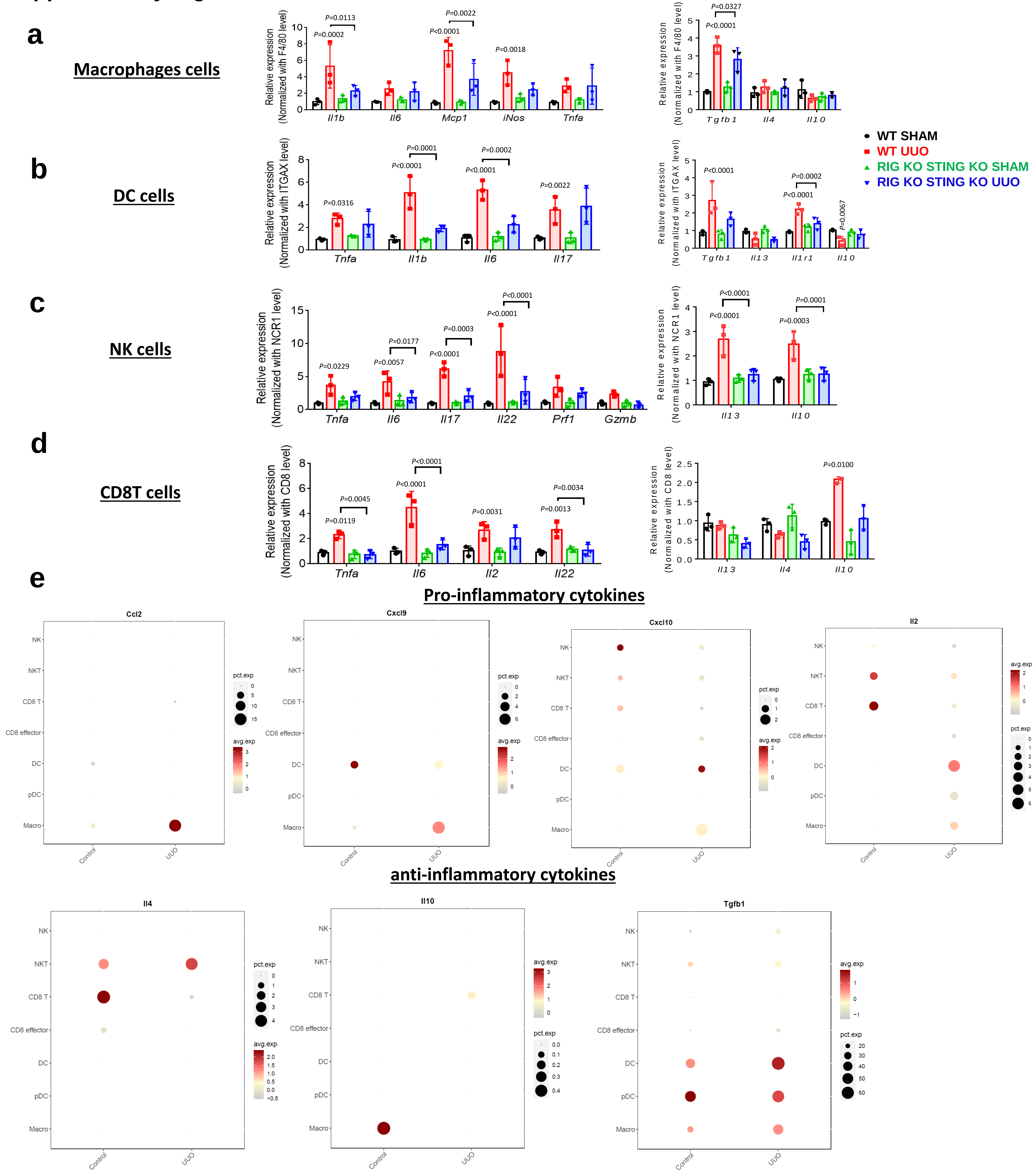

Supplementary Figure 14. Cytokines levels in isolated macrophages, DC, NK, and CD8T cells from kidneys of SHAM and UUO surgery mice.

(a) Relative mRNA levels of pro-inflammatory cytokines (*Il1b, Il6, Mcp1, iNos,* and *Tnfa)* and anti-inflammatory cytokines (*Tgfb1, Il4*, and *Il10*) in macrophage cells isolated from SHAM or UUO kidneys of WT and RIG KO STING KO mice (WT SHAM, black; WT UUO, red; RIG KO STING KO SHAM, green; RIG KO STING KO UUO, blue) (*n* = 3 in each). **(b)** Relative mRNA levels of pro-inflammatory cytokines (*Tnfa, Il1b, Il6,* and *Il17)* and anti-inflammatory cytokines *(Tgfb1, Il13, Ilr1,* and *Il10)* in Dendritic cells (DC) isolated from SHAM or UUO kidneys of WT and RIG KO STING KO mice (*n* = 3 in each). **(c)** Relative mRNA levels of pro-inflammatory cytokines (*Tnfa, Il6, Il17, Il22, Prf1,* and *Gzmb)* and anti-inflammatory cytokines *(Il13 and Il10)* in natural killer (NK) cells isolated from SHAM or UUO kidneys of WT and RIG KO STING KO mice (*n* = 3 in each). **(d)** Relative mRNA levels of pro-inflammatory cytokines (*Tnfa, Il6, Il2,* and *Il22) and* anti-inflammatory cytokines *(Il13, Il4, and Il10)* in CD8T cells isolated from SHAM or UUO kidneys of WT and RIG KO STING KO mice (*n* = 3 in each). Data are represented as mean ± s.e.m. and analyzed using a one-way ANOVA followed by Tukey post hoc test for multigroup (**a-d**). **(e)** Bubble plots showing the average gene expression levels for pro-inflammatory cytokines (*Ccl2, Cxcl9, Cxcl10*, and *Il2*) and anti-inflammatory cytokines (*Il4, Il10,* and *Tgfb1*) and percentage of expressing cells across all cell types from SHAM and UUO kidneys of WT and RIG KO STING KO mice. Source data are provided as a Source Data file.

Supplementary Figure 15

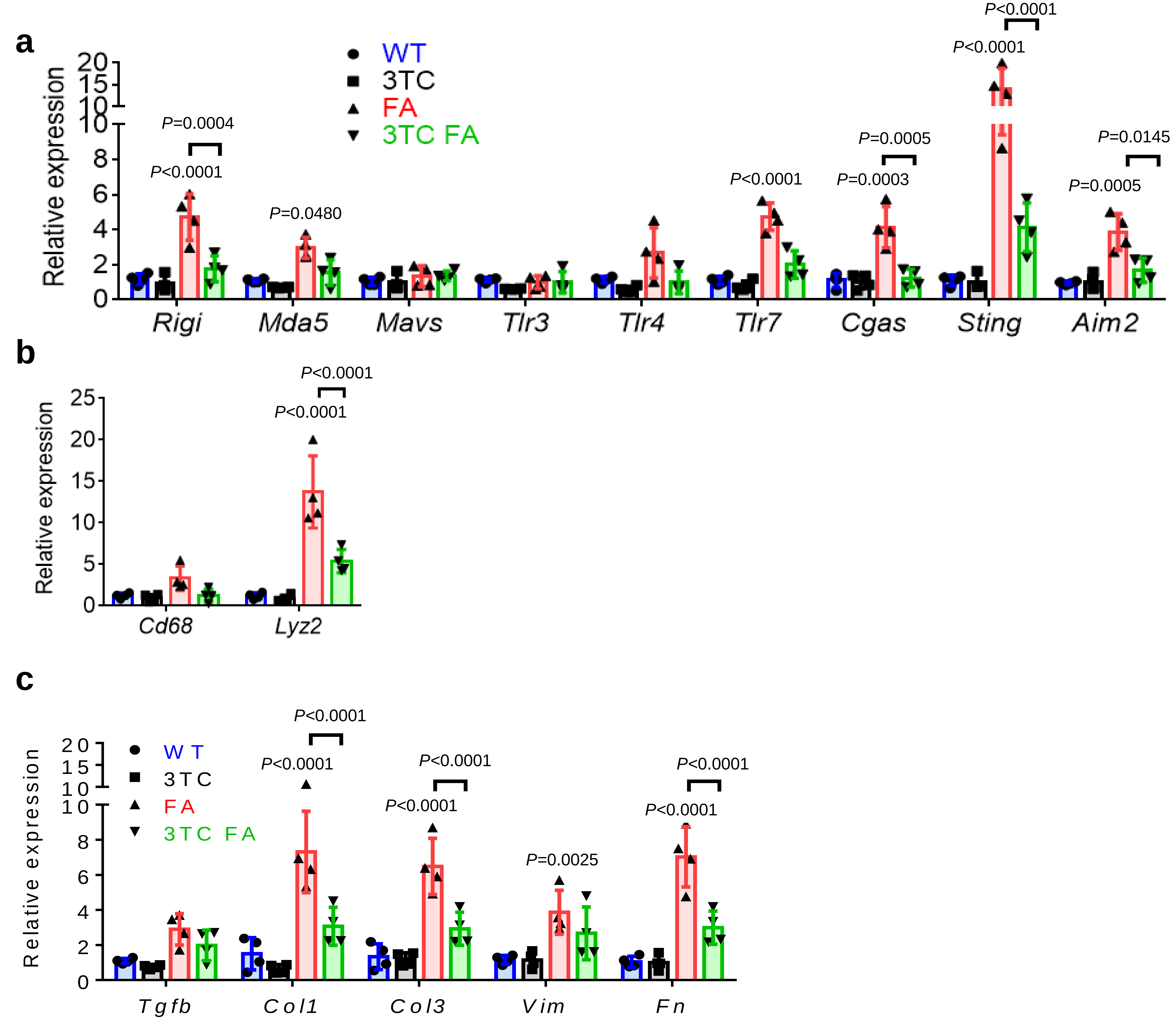

Supplementary Figure 15. The reverse transcriptase inhibitor Lamivudine (3TC) ameliorates fibroinflammation in FA-induced nephropathy.

(a) Relative RNA levels of cytosolic nucleic acid sensors (*Rigi, Mda5, Mavs, Tlr3, Tlr4, Tlr7, Cgas, Sting,* and *Aim2)* in kidneys of WT and FA mice treated with or without 3TC (WT, blue; 3TC, black; FA, red; 3TC FA, green)(*n* = 4 in each). **(b)** Expression of macrophage marker s (*Cd68* and *Lyz2)* in kidneys of WT and FA mice treated with or without 3TC (*n* = 4 in each). **(c)** Relative mRNA level of fibrosis markers (*Tgfb1, Col1, Col3, Vim,* and *Fn*) in kidneys WT and FA mice treated with or without 3TC (*n* = 4 in each). Data are represented as mean ± s.e.m. and analyzed using a two-way ANOVA followed by Tukey post hoc test for multigroup (**a-c**). Source data are provided as a Source Data file.

Supplementary Figure 16

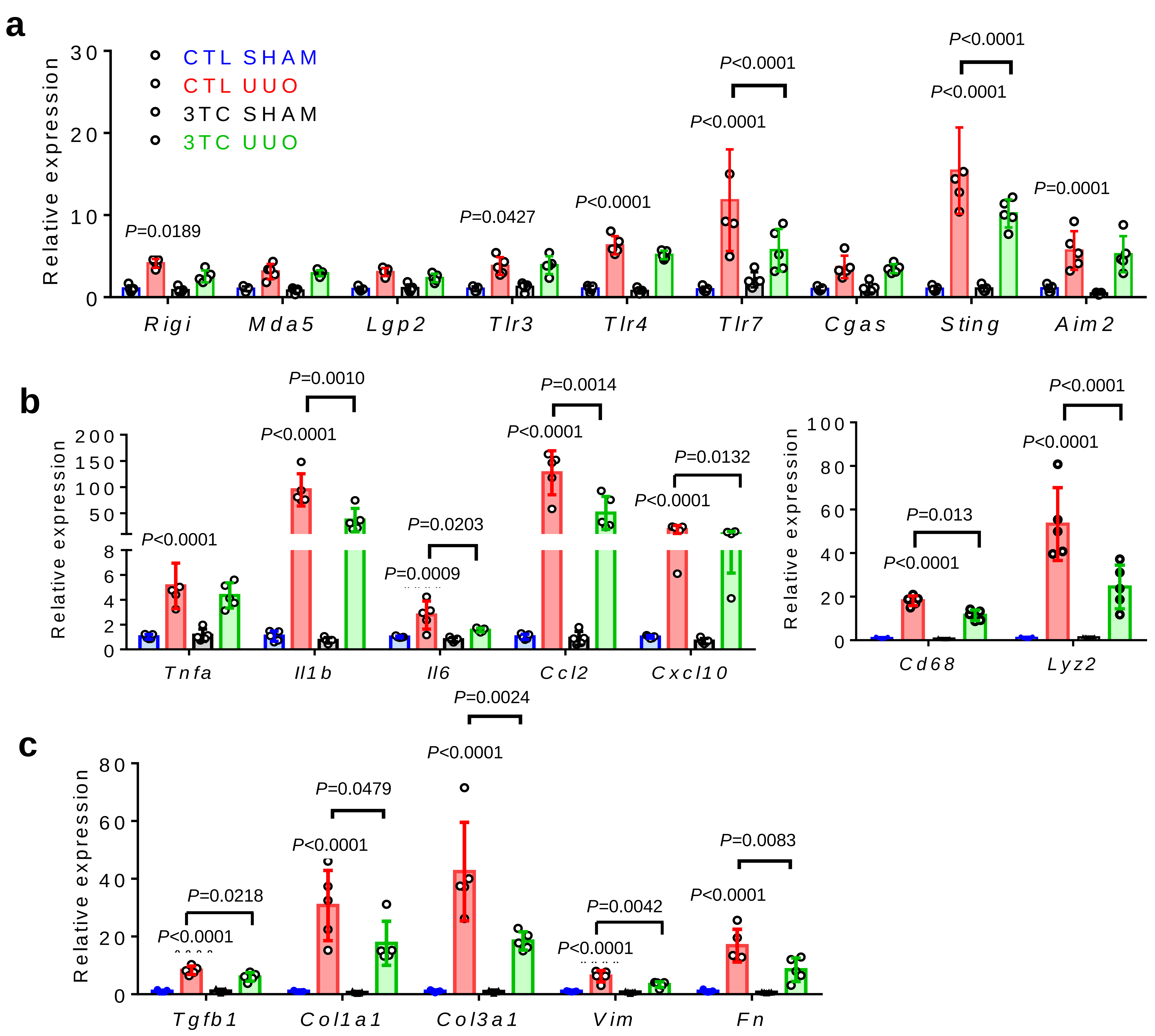

Supplementary Figure 16. Lamivudine (3TC) treatment reduced UUO-induced fibroinflammation in mice. **(a)** Relative mRNA levels of cytosolic nucleotide sensors (*Rigi, Mda5, Lgp2, Tlr3, Tlr4, TLr7, Cgas, Sting,* and *Aim2*) in kidneys of SHAM and UUO mice treated with or without 3TC (CTL SHAM, blue; CTL UUO, red; 3TC SHAM, black; 3TC UUO, green) (*n* = 5 in each). **(b)** Relative mRNA levels of proinflammatory cytokines (*Tnfa, Il1b, Il6, Ccl2,* and *Cxcl10*) and macrophages markers (*lyz2* and *Cd68*) in kidneys of SHAM and UUO mice treated with and without 3TC (*n* = 5 in each). **(c)** Relative RNA levels of profibrotic genes (*Tgfb1, Col1, Col3, Vim,* and *Fn*) in kidneys of SHAM and UUO mice treated with or without 3TC (*n* = 5 in each). Data are represented as mean ± s.e.m. and analyzed using a one-way ANOVA followed by Tukey post hoc test for multigroup (**a-c**). Source data are provided as a Source Data file.
