# Supplementary material for: Increased levels of endogenous retroviruses trigger fibroinflammation and play a role in kidney disease development
Source: Nat Commun. 2023 Feb 2;14:559. doi: 10.1038/s41467-023-36212-w (PMC9895454; doi:10.1038/s41467-023-36212-w)
Supplement: Supplementary file 3 — Description of Additional Supplementary Files [file 41467_2023_36212_MOESM3_ESM.pdf]

Description of Additional Supplementary Files

Supplementary Data 1: Demographics, clinical and histological characteristics of human kidney samples.

Supplementary Data 2: List of differentially expressed TEs in human kidney samples. A linear regression model was implemented to examine the association between TE expression and interstitial fibrosis, using age, gender, race, diabetes, hypertension, batch, RIN, duplication, mitochondrial percentage, unmapped reads and unique reads as covariates. A Benjamini-Hochberg (FDR) adjusted significance threshold of 0.05 was considered statistically significant.

Supplementary Data 3: List of differentially expressed full-length ERVs in human kidney samples.
A linear regression model was implemented to examine the association between ERV provirus expression and interstitial fibrosis, adjusted for age, gender, race, diabetes, hypertension, batch, RIN, duplication, mitochondrial percentage, unmapped reads, and unique reads. Benjamini-Hochberg (FDR) adjusted significance threshold of 0.05 was considered statistically significant.

Supplementary Data 4: List of differentially expressed TEs in UUO and FA mouse models of kidney disease.
DESeq2 was used to test for differential TE expression between control and FA/UUO groups with cutoff (Log2FC) >1 and FDR<0.05.

Supplementary Data 5: Correlation between ERVs, cell-fraction and nucleic acid sensors in human kidney samples.

Supplementary Data 6: List of TEs that showed correlation CpG methylation in human kidney samples.

Supplementary Data 7: List of primers used in this study.
