# Supplementary material for: Increased levels of endogenous retroviruses trigger fibroinflammation and play a role in kidney disease development
Source: Nat Commun. 2023 Feb 2;14:559. doi: 10.1038/s41467-023-36212-w (PMC9895454; doi:10.1038/s41467-023-36212-w)
Supplement: Supplementary file 5 — Reporting Summary [file 41467_2023_36212_MOESM5_ESM.pdf]

nature portfolio

Corresponding author(s): Katalin Susztak

Last updated by author(s): Jan 3, 2023

Reporting Summary

Nature Portfolio wishes to improve the reproducibility of the work that we publish. This form provides structure for consistency and transparency in reporting. For further information on Nature Portfolio policies, see our Editorial Policies and the Editorial Policy Checklist.

Statistics

For all statistical analyses, confirm that the following items are present in the figure legend, table legend, main text, or Methods section.

n/a	Confirmed	
[ ]	[x]	The exact sample size (*n*) for each experimental group/condition, given as a discrete number and unit of measurement
[ ]	[x]	A statement on whether measurements were taken from distinct samples or whether the same sample was measured repeatedly
[ ]	[x]	The statistical test(s) used AND whether they are one- or two-sided<br>*Only common tests should be described solely by name; describe more complex techniques in the Methods section.*
[ ]	[x]	A description of all covariates tested
[ ]	[x]	A description of any assumptions or corrections, such as tests of normality and adjustment for multiple comparisons
[ ]	[x]	A full description of the statistical parameters including central tendency (e.g. means) or other basic estimates (e.g. regression coefficient) AND variation (e.g. standard deviation) or associated estimates of uncertainty (e.g. confidence intervals)
[ ]	[x]	For null hypothesis testing, the test statistic (e.g. *F*, *t*, *r*) with confidence intervals, effect sizes, degrees of freedom and *P* value noted<br>*Give P values as exact values whenever suitable.*
[x]	[ ]	For Bayesian analysis, information on the choice of priors and Markov chain Monte Carlo settings
[x]	[ ]	For hierarchical and complex designs, identification of the appropriate level for tests and full reporting of outcomes
[ ]	[x]	Estimates of effect sizes (e.g. Cohen's *d*, Pearson's *r*), indicating how they were calculated

Our web collection on statistics for biologists contains articles on many of the points above.

Software and code

Policy information about availability of computer code

Data collection	qRT-PCR data was collected on Applied Biosystem ViiA7. Western Blot images were collected by Odyssey Fc. Fluorescent and bright field images were collected by Olympus BX43F, Absorbance signal was collected by plate reader BioTekSynergy H1.

Data analysis	FastQC v.0.11.2 (https://https://www.bioinformatics.babraham.ac.uk/projects/fastqc/)<br>TrimGalore.0.6.6 (https://www.bioinformatics.babraham.ac.uk/projects/trim_galore/)<br>For human RNA-seq analysis: STAR. 2.4.1d and for mice RNA-seq analysis: STAR. 2-7-8a (https://github.com/alexdobin/STAR)<br>HTSeq.0.12.4(https://htseq.readthedocs.io/en/release_0.11.1/history.html#version-0-6-1)<br>CIBERSORTx (https://https://cibersortx.stanford.edu/)<br>HervQuant.1.1 (https://unclineberger.org/vincentlab/resources/)<br>RSEM-1.3.0 (https://https://github.com/deweylab/RSEM)<br>R packages v4.1.1 (https://www.r-project.org/)<br>ggplot v2.3.3.5 (https://ggplot2.tidyverse.org/)<br>HTSeq v0.12.4 (https://https://htseq.readthedocs.io/en/master/)<br>DESeq v2.1.32.0 (https://bioconductor.org/packages/release/bioc/html/DESeq2.html)<br>SeSame v1.5.3 (https:// https://github.com/zwdzwd/sesame)<br>ImageJ v1.53 (https://imagej.nih.gov/ij) for Sirius red quantification.<br>Image Studio Lite Version 5.2 LICOR (https://www.licor.com/bio/image-studio-lite/d5)<br>Graphpad Prism 6 (https://www.graphpad.com/scientific-software/prism)

For manuscripts utilizing custom algorithms or software that are central to the research but not yet described in published literature, software must be made available to editors and reviewers. We strongly encourage code deposition in a community repository (e.g. GitHub). See the Nature Portfolio guidelines for submitting code & software for further information.

Data

Policy information about availability of data

All manuscripts must include a data availability statement. This statement should provide the following information, where applicable:

- Accession codes, unique identifiers, or web links for publicly available datasets
- A description of any restrictions on data availability
- For clinical datasets or third party data, please ensure that the statement adheres to our policy

Human RNA-seq data are available at Gene Expression Omnibus (GEO) with the accession code GSE115098 (https://www.ncbi.nlm.nih.gov/geo/query/acc.cgi?acc=GSE115098) and GSE173343 (https://www.ncbi.nlm.nih.gov/geo/query/acc.cgi?acc=GSE173343). Mouse FA model scRNA-seq data available at GEO with accession code GSE156686 (https://www.ncbi.nlm.nih.gov/geo/query/acc.cgi?acc=GSE156686) and can be viewed on SUSZTAK LAB kidney website (https:// https://susztaklab.com/visCello/). Mouse UUO scRNA-seq data is available at GEO with accession code GSE182256 (https://www.ncbi.nlm.nih.gov/geo/query/acc.cgi?acc=GSE182256) and can be viewed on SUSZTAK LAB website (https:// https://susztaklab.com/HK/scRNA/). The TE/ ERVs data generated in this study are provided in the Supplementary Information/Source Data file. Further information and requests for resources and reagents should be directed to and will be fulfilled by the lead contact: Katalin Susztak. Email: ksusztak@pennmedicine.upenn.edu

Human research participants

Policy information about studies involving human research participants and Sex and Gender in Research.

Reporting on sex and gender	For TE analysis, total 240 human kidney samples were used and gender ratio (Female:Male) was 94:146.<br>For full-length ERVs analysis, total 485 human kidney samples were used and gender ratio (Female:Male) was 185:300.
Population characteristics	Population characteristics for adult human kidney samples were provided in Supplemental Data 1. This table include information about Age, Gender, Ethnicity, background history (BMI, Hypertension, Diabetes, and Systolic Blood Pressure), renal function (Serum Albumin and eGFR), and Histology (Glomerulosclerosis and Interstitial Fibrosis).
Recruitment	Deidentified human kidney samples were obtained from the non-neoplastic portion of surgical nephrectomies via the Cooperative Human Tissue Network approved by the University of Pennsylvania Institutional Review Board. Laboratory data (including serum creatinine) and demographic and clinical information including age, sex, self-reported ethnicity, diabetes and hypertension status was collected from medical records by an honest broker, therefore no informed consent was obtained from the subjects.
Ethics oversight	The Human study was deemed exempted by institutional review board (IRB) of the University of Pennsylvania (exemption IV). Therefore, Consent was not obtained from participants and there was no participant compensation.

Note that full information on the approval of the study protocol must also be provided in the manuscript.

Field-specific reporting

Please select the one below that is the best fit for your research. If you are not sure, read the appropriate sections before making your selection.

[x] Life sciences [ ] Behavioural & social sciences [ ] Ecological, evolutionary & environmental sciences

For a reference copy of the document with all sections, see nature.com/documents/nr-reporting-summary-flat.pdf

Life sciences study design

All studies must disclose on these points even when the disclosure is negative.

Sample size	The primary cohort included 240 (for TE analysis and to profile CpG methylation) and 485 (for full-length ERV analysis) human kidney samples that were collected from the unaffected portion of surgical nephrectomies.<br>No sample size calculation was performed. For the animal experiment, sample size are determined based on the means and variation of previous pilot and published experiments (PMID: 30275566, 33998598, 34426578). For cell and biochemical data, we aimed to collect data from at least three biological replicates.
Data exclusions	No data were excluded in the study.
Replication	All attempts at replication were successful. Independent repeated times for each experiment were indicated in the figure legends.
Randomization	Mice were randomly allocated to between experimental groups.
Blinding	Blinding was not relevant with this type of analysis, we collected samples that were available to us. Investigators were blinded to allocation during experiments and outcome assessments.

Reporting for specific materials, systems and methods

We require information from authors about some types of materials, experimental systems and methods used in many studies. Here, indicate whether each material, system or method listed is relevant to your study. If you are not sure if a list item applies to your research, read the appropriate section before selecting a response.

Materials & experimental systems

n/a	Involved in the study	
☐	☒	Antibodies
☐	☒	Eukaryotic cell lines
☒	☐	Palaeontology and archaeology
☐	☒	Animals and other organisms
☒	☐	Clinical data
☒	☐	Dual use research of concern

Methods

n/a	Involved in the study	
☒	☐	ChIP-seq
☒	☐	Flow cytometry
☒	☐	MRI-based neuroimaging

Antibodies

Antibodies used	HERV-K (Novus, #H00002087-A01), RIG-I (CST, #3743), MDA5 (CST, #5321), STING (CST, #13647), pSTING (CST, #19781), cGAS (CST, #31659), pTBK1 (CST, #5483), TBK1 (CST, #3504), IRF3 (CST, #1190T), pIRF3 (CST, #37829), GAPDH (CST, #2118), ACTIN (Sigma, #A3854), IRF7 (CST, #4920), pIRF7 (CST, #24129),pP65 (CST, #3033), p65 (CST, #8242), FN (Abcam, #ab2413), IKKε (CST, #3416), pIKKε (CST, #8766) and SMA (Sigma, #A5228). HRP-conjugated secondary anti-rabbit (CST, #7074) and anti-mouse antibody (CST, #7076), IR-conjugated anti-mouse (CST, #5470S) and anti-rabbit (CST, #5151S) antibody were used as secondary antibodies for western blot. CD4 (Thermo, #36-0041-85 for mouse and #14-0049-82 for human), CD8 (Thermo, #36-0081-85 for mouse and #12-0088-80 for human), and F4/80 (Thermo, #MA191124) used for IHC. For viral RNA, we used dsRNA (SCICONS, #10010200) and LTL (Vector Lab, #FL-1321) for proximal tubules marker. Alexa Fluor 555(Invitrogen, #A31572) and Alexa Fluor 488 (Invitrogen, #21200) were used as secondary antibody and nuclei was stained with DAPI (Thermo, #62248).
Validation	All antibodies were previously validated by the manufacturer for given species and application. Validation statement are available at vendors' websites as follow:<br>HERV-K: https://www.novusbio.com/products/ervk2-antibody_h00002087-a01#datasheet<br>RIG-I: https://www.cellsignal.com/products/primary-antibodies/rig-i-d14g6-rabbit-mab/3743<br>MDA5: https://www.cellsignal.com/products/primary-antibodies/mda-5-d74e4-rabbit-mab/5321<br>STING: https://www.cellsignal.com/products/primary-antibodies/sting-d2p2f-rabbit-mab/13647<br>pSTING: https://www.cellsignal.com/products/primary-antibodies/phospho-sting-ser366-d7c3s-rabbit-mab/19781<br>cGAS: https://www.cellsignal.com/products/primary-antibodies/cgas-d3o8o-rabbit-mab-mouse-specific/31659<br>pTBK1: https://www.cellsignal.com/products/primary-antibodies/phospho-tbk1-nak-ser172-d52c2-xp-rabbit-mab/5483<br>TBK1: https://www.cellsignal.com/products/primary-antibodies/tbk1-nak-d1b4-rabbit-mab/3504<br>IRF3: https://www.cellsignal.com/products/primary-antibodies/irf-3-d6i4c-xp-rabbit-mab/11904<br>pIRF3: https://www.cellsignal.com/products/primary-antibodies/phospho-irf-3-ser386-e7j8g-xp-rabbit-mab/37829<br>GAPDH: https://www.cellsignal.com/products/primary-antibodies/gapdh-14c10-rabbit-mab/2118<br>ACTIN: https://www.sigmaaldrich.com/US/en/product/sigma/a3854<br>IRF7: https://www.cellsignal.com/products/primary-antibodies/irf-7-antibody/4920<br>pIRF7: https://www.cellsignal.com/products/primary-antibodies/phospho-irf-7-ser437-438-d6m2i-rabbit-mab-mouse-specific/24129<br>pP65: https://www.cellsignal.com/products/primary-antibodies/phospho-nf-kb-p65-ser536-93h1-rabbit-mab/3033<br>P65: https://www.cellsignal.com/products/primary-antibodies/nf-kb-p65-d14e12-xp-rabbit-mab/8242

FN: https://www.abcam.com/fibronectin-antibody-ab2413.html
IKKε: https://www.cellsignal.com/products/primary-antibodies/ikke-d61f9-xp-rabbit-mab/3416
pIKKε :https://www.cellsignal.com/products/primary-antibodies/phospho-ikke-ser172-d1b7-rabbit-mab/8766
SMA: https://www.sigmaaldrich.com/US/en/product/sigma/a5228
HRP-conjugated secondary anti-rabbit: https://www.cellsignal.com/products/secondary-antibodies/anti-rabbit-igg-hrp-linked-antibody/7074
HRP-conjugated secondary anti-mouse: https://www.cellsignal.com/products/secondary-antibodies/anti-rabbit-igg-hrp-linked-antibody/7076
IR-conjugated anti-mouse: https://www.cellsignal.com/products/secondary-antibodies/anti-mouse-igg-h-l-dylight-680-conjugate/5470
IR-conjugated anti-rabbit: https://www.cellsignal.com/products/secondary-antibodies/anti-rabbit-igg-h-l-dylight-800-4x-peg-conjugate/5151
CD4 for mouse: https://www.thermofisher.com/antibody/product/CD4-Antibody-clone-GK1-5-Monoclonal/36-0041-85
CD4 for Human: https://www.thermofisher.com/antibody/product/CD4-Antibody-clone-RPA-T4-Monoclonal/14-0049-82
CD8 for mouse: https://www.thermofisher.com/antibody/product/CD8a-Antibody-clone-53-6-7-Monoclonal/36-0081-85
CD8 for human: https://www.thermofisher.com/antibody/product/CD8a-Antibody-clone-RPA-T8-Monoclonal/12-0088-80
F4/80: https://www.thermofisher.com/antibody/product/F4-80-Antibody-/MA1-91124
dsRNA: https://www.labome.com/product/SCICONS/10010200.html
LTL: https://vectorlabs.com/products/glycobiology/fluorescein-lotus-tetragonolobus-lectin-ltl
Alexa Flour555: https://www.thermofisher.com/antibody/product/Donkey-anti-Rabbit-IgG-H-L-Highly-Cross-Adsorbed-Secondary-Antibody-Polyclonal/A-31572
Alexa Flour 488: https://www.thermofisher.com/antibody/product/Chicken-anti-Mouse-IgG-H-L-Cross-Adsorbed-Secondary-Antibody-Polyclonal/A-21200
DAPI: https://www.thermofisher.com/order/catalog/product/62248

Eukaryotic cell lines

Policy information about cell lines and Sex and Gender in Research

Cell line source(s)	Primary TECs were isolated from mouse kidneys. HKC-8 cells were obtained from Dr. Lorraine C. Racusen from Department of Pathology, The Johns Hopkins University School of Medicine, Baltimore, MD 21205, USA
Authentication	Culture protocol for primary TECs cells were previously validated in our lab (PMID: 30226866, 33441424, 34426578) by evaluating PT cell specific gene using qRT-PCR and polarization was validated by confocal microscopy . HKC-8 were previously validated by Dr. Racusen using morphology, brush border enzyme activities, amyloglucosidase transport, and NaK- adenosine triphosphatase activity assays (PMID: 9042817) and in lab by morphology and PT cell specific gene expression using qRT-PCR (PMID: 25419705).
Mycoplasma contamination	Primary TECs were not tested for mycoplasma contamination and HKC-8 cells were negative for mycoplasma when tested.
Commonly misidentified lines (See ICLAC register)	This study did not involve misidentified lines.

Animals and other research organisms

Policy information about studies involving animals; ARRIVE guidelines recommended for reporting animal research, and Sex and Gender in Research

Laboratory animals	All animal experiments were reviewed and approved by the Institutional Animal Care and Use Committee of University of Pennsylvania and were performed in accordance with the institutional guidelines (protocol #804138). All mice were maintained under SPF conditions with ambient temperature 20-22, humidity 50-70% and a 12/12h light/dark cycle and fed with standard mouse diet and water ad libitum. 6 to 8 week old C57BL/6 wild type mice were used in the study. Both male and female mice were used in random fashion. RIG KO mice were purchased from Jackson Lab (Stock#46070) and 6 to 8 week old RIG KO mice were used in the study. STING KO mice were purchased from Jackson Lab (Stock#025805) and 6 to 8 week old STING KO mice were used in the study. DNMT1 F/F mice were obtained from Mutant Mouse Regional Resource Center (MMRRC_014114 UCD). Tet O Cre (TRE-Cre (stock #006234) and Pax8rtTA mice (Stock #007176) were purchased from Jackson Laboratory. 6 to 8 week old Pax8rtTA-Tet'o'Cre-DNMT1 F/F were used in this study.
Wild animals	This study did not involve wild animals.
Reporting on sex	Both Male and females mice were used in this study. This is not gender biased study.
Field-collected samples	This study did not involve samples collected from the field.
Ethics oversight	All animal experiments were reviewed and approved by the Institutional Animal Care and Use Committee of University of Pennsylvania and were performed in accordance with the institutional guidelines.

Note that full information on the approval of the study protocol must also be provided in the manuscript.
